# Electroacupuncture Promotes Proliferation of Amplifying Neural Progenitors and Preserves Quiescent Neural Progenitors from Apoptosis to Alleviate Depressive-Like and Anxiety-Like Behaviours

**DOI:** 10.1155/2014/872568

**Published:** 2014-02-26

**Authors:** Liu Yang, Na Yue, Xiaocang Zhu, Qiuqin Han, Bin Li, Qiong Liu, Gencheng Wu, Jin Yu

**Affiliations:** ^1^Institute of Acupuncture Research, WHO Collaborating Centre for Traditional Medicine, Department of Integrative Medicine and Neurobiology, State Key Lab of Medical Neurobiology, Institutes of Brain Science, Shanghai Medical College, Fudan University, Shanghai 200032, China; ^2^Department of Anatomy, Histology and Embryology, Shanghai Medical College, Fudan University, Shanghai 200032, China

## Abstract

The present study was designed to investigate the effects of electroacupuncture (EA) on depressive-like and anxiety-like behaviours and neural progenitors in the hippocampal dentate gyrus (DG) in a chronic unpredictable stress (CUS) rat model of depression. After being exposed to a CUS procedure for 2 weeks, rats were subjected to EA treatment, which was performed on acupoints Du-20 (Bai-Hui) and GB-34 (Yang-Ling-Quan), once every other day for 15 consecutive days (including 8 treatments), with each treatment lasting for 30 min. The behavioural tests (i.e., forced swimming test, elevated plus-maze test, and open-field entries test) revealed that EA alleviated the depressive-like and anxiety-like behaviours of the stressed rats. Immunohistochemical results showed that proliferative cells (BrdU-positive) in the EA group were significantly larger in number compared with the Model group. Further, the results showed that EA significantly promoted the proliferation of amplifying neural progenitors (ANPs) and simultaneously inhibited the apoptosis of quiescent neural progenitors (QNPs). In a word, the mechanism underlying the antidepressant-like effects of EA is associated with enhancement of ANPs proliferation and preserving QNPs from apoptosis.

## 1. Introduction

Acupuncture, particularly electroacupuncture (EA), is well-known for its profound therapeutic value with fewer side effects in many diseases [1, 2]. A number of studies have manifested that acupuncture is an effective remedy for depression and it may be as effective as antidepressant drugs [3]. Previous studies demonstrate that EA, just like other antidepressant strategies, such as electroconvulsive seizure (ECS) and selective serotonin reuptake inhibitors (SSRIs), increases neurogenesis in the adult hippocampus and this effect is thought to contribute, in part, to the actions of these treatments in rodent [4–6]. However, the precise cellular basis mediating their antidepressant-like effects has not been fully characterized.

Two major subclasses of neural progenitors (NPs) have been characterized in the dentate gyrus (DG) of the adult hippocampus based on the expression of phenotypic marker proteins. The first class is called quiescent neural progenitors (QNPs), which has a radial glia-like morphology and carry stem cell properties, characterized by expression of glial fibrillary acid protein (GFAP) and a relatively low rate of proliferative activity [7–12]. The second class of NPs is considered the progeny of QNPs and named amplifying neural progenitors (ANPs). It is GFAP-negative and has a higher rate of mitotic activity [9, 10, 13, 14]. For their different mitotic rates, only a small fraction of QNPs can be labelled with a nucleotide analog 5-bromo-2-deoxyuridine (BrdU) after a short pulse, but ANPs can be labelled with BrdU with high frequency [8, 10]. ANPs and QNPs in the hippocampal DG also express the transcription factor Sox2, which is considered a marker for NPs in neurogenic regions [13, 15–17].

Two studies have reported that antidepressants, such as ECS and fluoxetine, exhibited different effects on the proliferation of ANPs and QNPs in adult rodent hippocampus [5, 10]. Although our previous study has demonstrated that EA restored the impaired proliferation of NPs within the DG in a rat model of depression [4], the accurate cellular mechanisms underlying the antidepressant-like effect of EA still require further investigation. To address this issue, we explored the effect of EA on the proliferation of the QNP and ANP subclasses in DG of rat hippocampus.

Furthermore, some studies have provided insight into the potential role of neural apoptosis in the cellular hypothesis of depression [18, 19]. And the inhibition of neural apoptosis maybe mediated the effect of many antidepressants [20–25]. But all relative studies seldom focus on the apoptosis of NPs although they are also undergoing apoptosis [26]. We have observed the astroglial atrophy in a depression model of rats [27], so we sought to simultaneously assess whether the apoptosis of NPs is involved in the antidepressant-like effects of EA in rats.

The results showed that chronic unpredictable stress (CUS) induced depressive-like and anxiety-like behaviours in rats and simultaneously exerted differential influence on the subclasses of NPs; that is, it enhanced the apoptosis of QNPs slightly and impaired the proliferation of both ANPs and QNPs in DG of adult rodent hippocampus. Conversely, EA alleviated depressive-like and anxiety-like behaviours in the rat CUS model of depression, restored the proliferation of ANPs, and suppressed the apoptosis of QNPs to preserve the NPs in hippocampus.

## 2. Materials and Methods

### 2.1. Animals

Sprague Dawley rats (male, 220 g) from the Experimental Animal Centre of Fudan University were group-housed (4 per cage) on a 12 h light/dark cycle at 18°C~22°C with food and water available *ad libitum*, unless noted otherwise. The rats were allowed to adjust to their new surroundings for 1 week prior to CUS. The experimental protocol used in this study was in strict accordance with the National Institutes of Health Guide for the Care and Use of Laboratory Animals and approved by the Experimental Animal Ethics Committee of Shanghai Medical College, Fudan University (20120302-107).

### 2.2. CUS Procedure

The CUS model was first described by Katz [28] and has been validated as one of the most relevant models of depression. In this procedure, rats were exposed (in random order) to a variety of severe stressors over a long period of time. It has been well established that CUS can cause neurobehavioural disturbances in rodents and mimic symptoms of depression [29]. During the modified CUS procedure, 7 types of stressors were performed, including (1) 40 h water deprivation, (2) 40 h food deprivation, (3) light-dark cycle reversal, (4) 5 min hot environment (40°C), (5) 5 min swimming in cold water (5°C), (6) cage shake (30 min), and (7) wet cage. The stressors were applied in a random order for 4 weeks. The CUS rats were singly-housed during the stress period, while the control animals were group-housed (4 per cage) throughout the study.

### 2.3. EA Delivery

During EA treatment, the rat was moderately bound by a piece of self-made clothing and hung approximately 0.15 m high so that the movement of its body was restrained, but its head could move freely. A pair of stainless steel needles connected with the output terminals of an EA apparatus (HANS Electronic Apparatus, LH202H) penetrated into the acupoints “Bai-Hui” (Du-20, located above the apex auriculate, on the midline of the head) and “Yang-Ling-Quan” (GB-34, anterior and inferior to the head of the fibula), followed by a 30 min electric stimulation (2∖100 Hz, 0.3 mA). The Model group was bound and hung for 30 min. The EA was administered every other day for 15 days (8 times), starting on day 15.

### 2.4. Behavioural Testing

**Figure 1:**
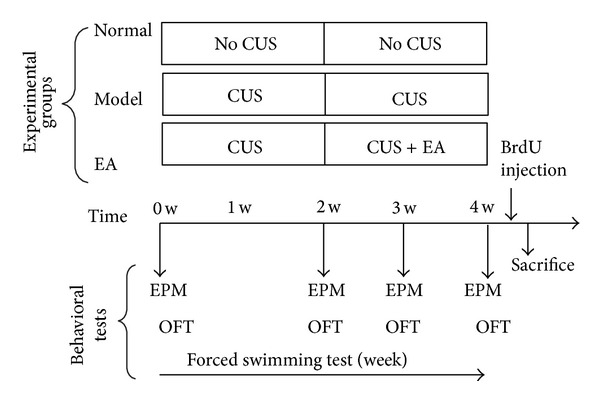
Animal groups and schematic representations of the behaviour testing procedure. The rats were randomly divided into three groups (*n* = 8 per group): Normal (a naïve unchallenged group without any stress and any treatment), Model (rats which were exposed to 4-week CUS), and EA (model rats which received EA treatment in the last two weeks of the CUS procedure). For the latter 2 groups, 4-week CUS with/without 2-week EA treatment was performed. The forced swimming test (FST) was performed at the end of every week. The elevated plus-maze (EPM) test and the open field test (OFT) were performed before the CUS, at the end of week 2, week 3, and week 4. For analysis of cell proliferation, another batch of the rats, which was also divided into three groups and subjected to the same procedure, received a single injection of BrdU (200 mg kg1 i.p.) on the first day of the week 5 and was sacrificed (S) 24 h after BrdU administration.

The following tests were employed to assess the depressive-like and anxiety-like behaviours of the experimental rats (Figure 1).

The forced swimming test (FST) was performed once a week. This test was widely used to detect the antidepressant-like effects in rodents [30]. The rat was forced to swim individually in a glass cylinder (30 cm in diameter) containing 25 cm of tap water (25 ± 1°C). At first, the rats were placed in the water for 15 min and retested for another 5 min after 24 h. The rats were subjected to a 5 min retest every week. After the test, the rats were dried with a towel and returned to their home cages. The total duration of immobility and climbing during the first 5 min of the swimming session was recorded and scored by an observer who was blind to the groups of animals. The rat was judged to be immobile when it floated without struggling and making only those movements necessary to keep its head above the water. Conversely, climbing was determined when the rat was vertically climbing the walls of the cylinder. A decrease in the duration of immobility or an increase in climbing time is an indicative of the antidepressant effect.

Each rat was subjected to the elevated plus-maze (EPM) test prior to CUS and at the end of weeks 2, 3, and 4. The EPM was used to assess the anxiety-like behaviour of the rat, as described previously [31]. The EPM consisted of two open arms (50 × 10 cm^2^) crossed with two similar closed arms and had walls of 40 cm in height. A 5 min session was used for each rat to determine (1) percentage of open/total arm entries and (2) percentage of time spent in the open/total arms. After testing each animal, the apparatus was cleaned with 1% acetic acid to prevent olfactory cues from affecting the behaviour of the subsequently tested rats.

The open field test (OFT) was applied to analyse the locomotion and exploration of the rats, as previously described [32]. The open field test was performed in a 100 × 100 cm^2^ box with 40 cm plastic walls, which was illuminated with a 60 W bulb hanging 90 cm above the centre of the field. The animals were individually placed into the centre facing the same direction. Each rat was recorded for 5 min to monitor the distance travelled and the number of rearings (defined as the animal standing upright on its hind legs). After testing each animal, the apparatus was cleaned with 1% acetic acid to remove olfactory cues. The OFT was performed before the CUS and at the end of weeks 2, 3, and 4.

### 2.5. BrdU Injection

Another batch of animals was exposed to CUS procedure only or with EA treatment, which was performed strictly as mentioned above, for the purpose of immunohistochemical analysis. To label proliferating NPs (S phase of mitosis), the rats were given 5-bromo-2-deoxyuridine (BrdU, 200 mg/kg, i.p., Sigma-Aldrich, USA) 24 h before perfusion.

### 2.6. Perfusion and Brain Section Preparation

The rats were anaesthetised with chloralic hydras (8%, 2 mL per rat) and perfused through the ascending aorta with 200 mL of PBS (0.1 M, PH = 7.4), followed by 300 mL 4% paraformaldehyde (PFA, in 0.1 M PBS, PH = 7.4). The brains were separated and postfixed in 4% paraformaldehyde at 4°C overnight and immersed in 20% sucrose (4% PFA as solvent) followed by 30% sucrose (in 0.1 M PBS). The brain samples were cut into 30 *μ*m thick sections through the entire DG on a freezing microtome (CM1850, Leica Microsystems, Wetzlar, Germany) and then stored in cryoprotectant (30% ethylene glycol, 30% sucrose, 0.1 M PBS, pH 7.4) at −20°C until use.

### 2.7. Immunohistochemistry

BrdU immunohistochemistry was carried out on free-floating sections as described previously [33]. The sections were incubated for 30 min in 2 N HCl at 37°C and then 10 min in boric acid. After washing for 5 × 10 min in PBS (0.01 M, PH = 7.4), the sections were incubated in blocking buffer (1% BSA and 4% horse serum in 0.3% Triton X-100) for 1 h. The sections were then incubated with sheep anti-BrdU (1 : 100, Abcam, UK, in 0.3% Triton X-100) at 4°C overnight. Next, the sections were incubated in a secondary antibody solution (donkey anti-sheep, 1 : 200 and R-phycoerythrin labeled, Jackson ImmunoResearch, USA, in 0.3% Triton X-100) in the dark for 1 h at 37°C. After 5 × 10 min washes in PBS (0.01 M, PH = 7.4), the sections were coverslipped and then examined under a fluorescence microscope.

The BrdU and GFAP double immunofluorescent staining or GFAP and Sox2 double immunofluorescent staining were performed as aforementioned protocol, except for the antibodies for GFAP (mouse anti-GFAP, 1 : 500, Neomarker, USA, goat anti-mouse, Alexa 488 labelled, 1 : 200, Invitrogen, USA, or goat anti-mouse, Alexa 594 labelled, 1 : 200, Invitrogen, USA) and Sox2 (rabbit anti-Sox2, 1 : 200, Sigma-Aldrich, USA, and goat anti-rabbit, Alexa 488 labelled, 1 : 200, Invitrogen, USA). In the first kind of double staining, BrdU^+^/GFAP^−^ cells correspond to the proliferating ANPs and those BrdU^+^/GFAP^+^ cells to the proliferating QNPs. In the second kind, GFAP^+^/Sox2^+^ cells correspond to QNPs, GFAP^−^/Sox2^+^ cells correspond to the ANPs, and Sox2^+^ cells correspond to the NPs in hippocampus.

The numbers of single or double-stained cells in the DG on one brain section were counted. At least three brain sections containing DG were selected from one brain for immunostaining. The mean for each rat was calculated and 8 brains were selected to represent one group.

### 2.8. Hoechst Staining

Hoechst 33342, which stains cellular DNA, is a fluorescent dye that has been widely used for analysis of nuclear morphology, for example, nuclear condensation and fragmentation in cultured neural cells and tissue section [34–36]. The death of NPs was then evaluated by fluorescence microscopy measurement of nuclear condensation and fragmentation after Hoechst 33342 staining combined with the above-mentioned GFAP and Sox2 double staining. Brain sections previously stained for GFAP and Sox2 were incubated for 5 minutes on the microscope slide with nuclear dye Hoechst 33342 (10 *μ*g/mL in PBS, Molecular Probes, Eugene, Ore). The slides were then rinsed 3 times with PBS for 5 minutes and coverslipped.

The apoptosis % of some specific cell population was calculated as the number of the apoptotic cells divided by the total number of this cell population. As mentioned above, the cell populations were differentiated by special cell markers, such as GFAP and Sox2. Briefly, the denominator is the number of some specific types of cells, and the numerator is the apoptotic number of these specific types. For every section, the total number of cells in the DG was calculated by counting the Hoechst-positive cells in the DG zone of a spliced photo (obtained by Nikon A1 Widefield Fluorescence Laser Scan Confocal Microscope, which ensured the total DG zone included in the photo) and similarly, total number of NSCs by Hoechst^+^/Sox2^+^, QNPs by Hoechst^+^/Sox2^+^/GFAP^+^, and ANPs by Hoechst^+^/Sox2^+^/GFAP^−^. Two or three brain sections at fontanel −3.9 mm were selected from one brain (to calculate the mean for each rat) and 5 brains were selected to represent one group.

### 2.9. Statistical Analysis

The data are analysed using one-way or two-way ANOVA or *t*-test and are presented as the means ± S. E. M. The statistical significance level was set as  *P* < 0.05.

## 3. Results and Discussion

### 3.1. Results

#### 3.1.1. CUS Induces Depressive-Like and Anxiety-Like Behaviours

**Figure 2:**
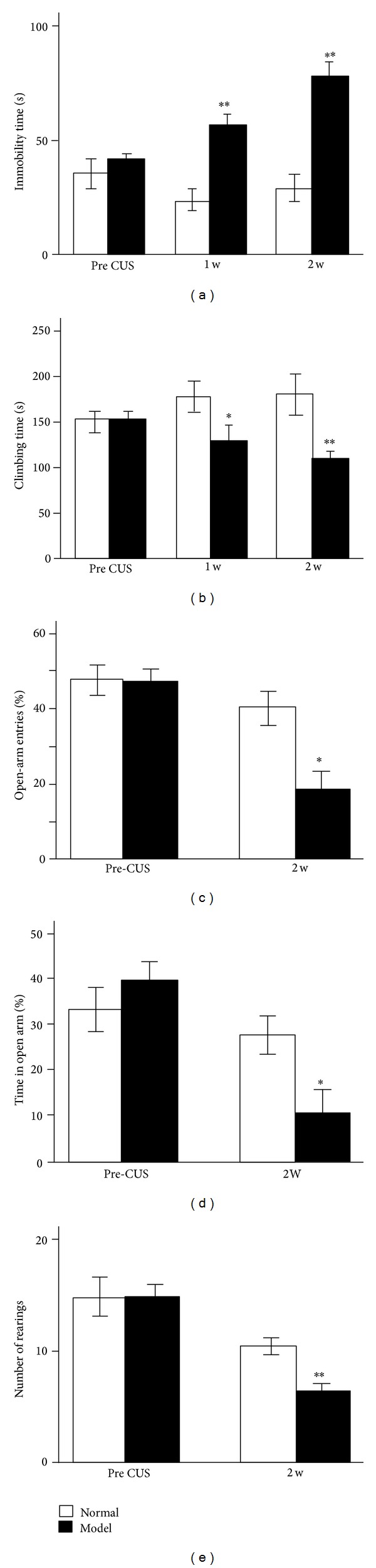
Stressed rats (Model group) show significantly depressive-like and anxiety-like behaviours after 2-week CUS exposure. (a) The immobility time and (b) the climbing time in forced swimming test are expressed in seconds. (c) Percentage of open-arm entries and (d) percentage of time spent in open arms during a 5 min test on the elevated plus-maze. (e) The number of rearing during the open field test. The results are expressed as the mean ± SEM. *P < 0.05 and **P < 0.01, versus Normal group.

Two weeks after CUS, the stressed rats exhibited significant depressive-like behaviours in the FST (i.e., increased immobility and decreased climbing,  *P* < 0.01, Figures 2(a) and 2(b)), anxiety-like behaviours in the EPM (i.e., a significantly lower preference for the open arms, Figures 2(c) and 2(d)), and lower exploratory behaviour in OFT (i.e., decreased rearing without changing total distance travelled, Figure 2(e)). These results indicated that the rat model of depression can be induced by CUS exposure.

#### 3.1.2. EA Reverses the Depression-Like Behaviours

**Figure 3:**
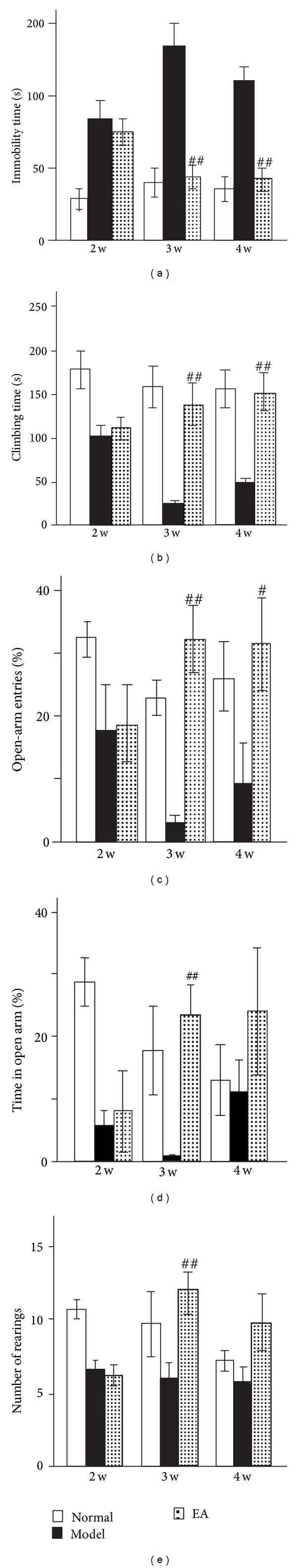
EA alleviates the CUS-induced depressive-like and anxiety-like behaviours. One- or two-week EA significantly decreases the immobility time (a) and increases the climbing time (b) in the forced swimming test. (c) One-week EA treatment significantly elevates the open-arm entries but not two-week EA treatment. (d) Stressed rats spend more time in the open arms in the elevated plus-maze test after one-week EA treatment but not two-week EA treatment. (e) One-week EA treatment reverses the declined rearing number of the stressed rats in the open field test. The results are expressed as the mean ± SEM. ^#^ *P* < 0.05 and ^##^ *P* < 0.01, versus Model group.

The EA group showed a significantly decreased immobility time in the FST compared with the Model group after one- or two-week treatment (*P* < 0.01, Figure 3(a)). Correspondingly, EA also significantly restored the decreased climbing time after two-week treatment (*P* < 0.01, Figure 3(b)).

After one- or two-week administration in CUS rats, EA could partly restore the percentage of open-arm entries and time spent in the open arms to a certain extent in the EPM test (at the end of week 3, *P* < 0.01, resp., and at the end of week 4, *P* < 0.05, *P* > 0.05, resp., Figures 3(c) and 3(d)).

In the OFT, compared with the Model group, the EA group also showed enhanced exploratory behaviour, that is, increased rearing only after one-week treatment (at the end of week 3, *P* < 0.01 and at the end of week 4, *P* = 0.111, Figure 3(e)).

#### 3.1.3. EA Promotes the Proliferation of ANPs but Not QNPs

**Figure 4:**
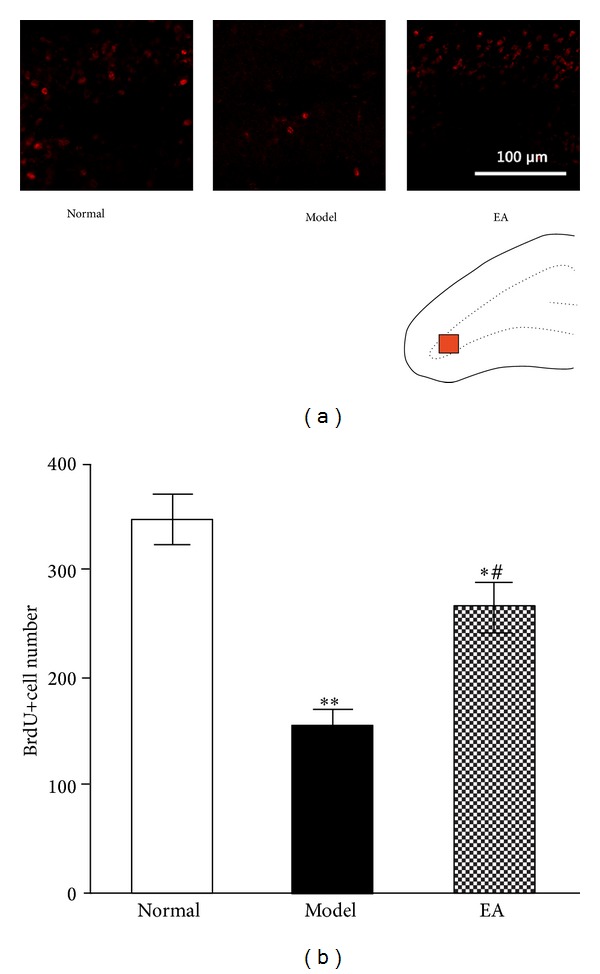
Proliferation study. EA increases the proliferative cells in the DG of the stressed rats. (a) Representative microphotographs showing BrdU-positive cells (administered 24 h before killing) in DG of rats in different groups. The red square in the schematic drawing indicates the regions of microphotographs shown in (a). Scale bar corresponds to 100 *μ*m. Bar gram in panel (b) depicts (mean ± SE) the quantification of the proliferation study showing the number of BrdU-positive cells in 3 experimental groups. *n* = 5, *P < 0.05, and **P < 0.01, versus Normal group; ^#^ *P* < 0.05, versus Model group.

As shown in Figure 4, most BrdU-positive cells were located in the subgranular zone (SGZ) of the DG. After 4-week exposure to CUS, the BrdU-positve cells in the DG of stressed rats were significantly decreased in comparison with normal rats (*P* < 0.01, Figure 4). The results intimated that the CUS significantly inhibited the proliferation of hippocampal NPs. By contrast, repeated EA treatments significantly upregulated the number of dividing NPs (BrdU-positive cells) in the hippocampus of stressed rats (*P* < 0.05, Figure 4), which was consistent with the behavioural tests.

**Figure 5:**
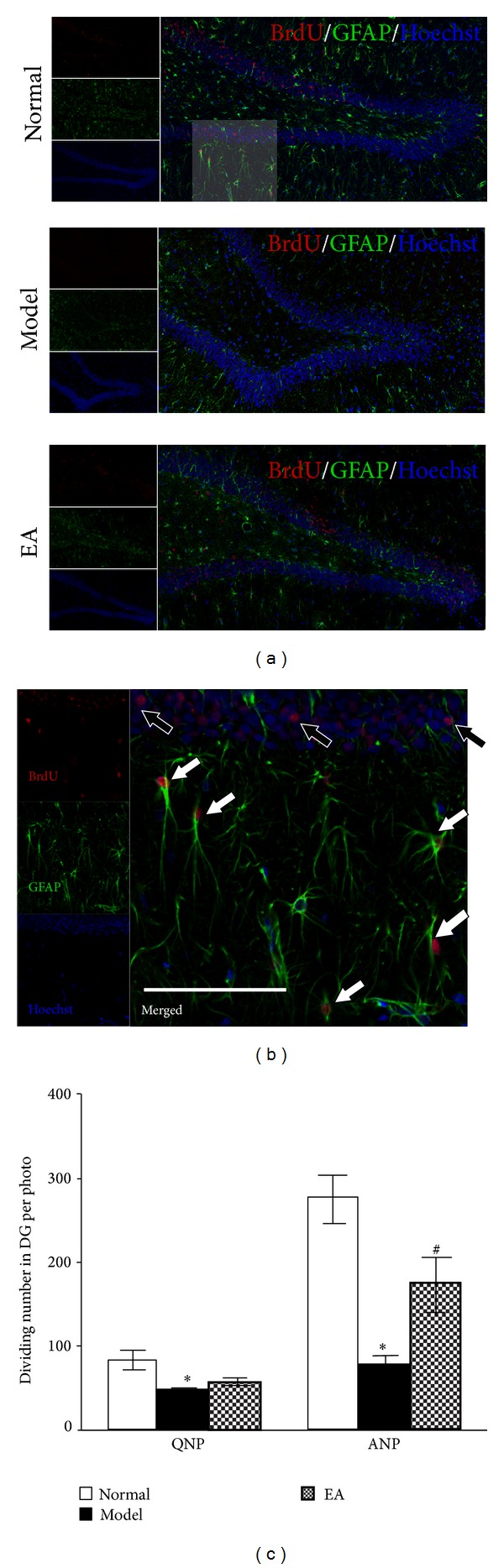
EA reverses the inhibition of ANPs proliferation in the DG of the stressed rats. (a) Representative triple-stained sections in the DG: BrdU (red), GFAP (green), and Hoechst (blue). The translucent square in the Normal figure is magnified in (b) to point out the division of quiescent neural progenitors (QNPs) and amplifying neural progenitors (ANPs). The BrdU^+^/GFAP^−^ cells (black arrows) are considered the proliferating ANPs and those BrdU^+^/GFAP^+^ cells (white arrows) the proliferating QNPs. Scale bar represents 100 *μ*m. Bar gram in panel (c) depicts (mean ± SE) the quantification of the dividing cells belonging to different subtypes of NPs in the whole DG. The proliferations of QNPs and ANPs are both decreased by CUS, and EA upregulates the ANP proliferation in stressed rats. *n* = 5 and *P < 0.05, versus Normal group; ^#^ *P* < 0.05, versus Model group.

We further defined the different response of the subclass of NPs to stress EA. As mentioned above, the different pattern of GFAP expression in NPs was used to distinguish between the ANPs and QNPs combined with BrdU labeling. As described in Figure 5, CUS significantly decreased the number of dividing QNPs (BrdU^+^/GFAP^+^) and dividing ANPs (BrdU^+^/GFAP^−^) (*P* < 0.05, resp., Figure 5). Nevertheless, EA only increased the number of dividing ANPs (BrdU^+^/GFAP^−^) but not dividing QNPs (BrdU^+^/GFAP^+^) (*P* < 0.05, Figure 5). These results suggested that CUS significantly decreased the proliferation of both ANPs and QNPs, but EA only partly restored the decreased proliferation of ANPs but not QNPs.

#### 3.1.4. EA Inhibits Apoptosis of QNPs but Not ANPs

**Figure 6:**
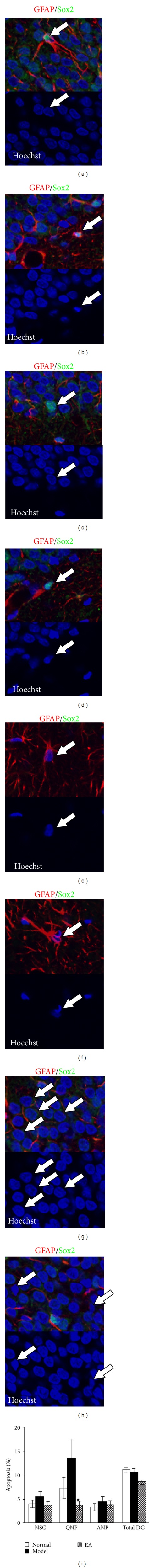
EA relieves the apoptosis of QNPs in DG of the stressed rats. ((a)–(h)) Representative GFAP (red), Sox2 (green), and Hoechst (blue) triple-stained sections show that typical cells are undergoing apoptosis or not in different types. The Hoechst staining is below the merged triple staining in every figure to show the typical apoptotic morphology of the nucleus (pyknosis, deep into dense) or normal nucleus (without any nuclear condensation and fragmentation). (a) A normal QNP (arrow). (b) An apoptotic QNP (arrow). (c) A normal ANP (arrow). (d) An apoptotic ANP (arrow). (e) A normal astrocyte (arrow). (f) An apoptotic astrocyte (arrow). (g) Normal granular cells in GCL (arrows). (h) Apoptotic granular cells in GCL (arrows). Bar gram in panel (i) depicts (mean ± SE) the quantification of the different types of the apoptotic cells in the whole DG. EA protects the stressed rats when they exhibits an antiapoptotic effect on the QNPs in DG. *n* = 5 and ^#^ *P* < 0.05, versus Model group.

Apoptosis is common in the hippocampal neurogenic niche, as vast amounts of newborn cells die [37]. In other words, NPs underwent not only division but also apoptosis during its whole life. As we know, a cell that is undergoing apoptosis demonstrates nuclear condensation, which can be detected by staining with Hoechst 33342. In this experiment, the number of apoptotic cells in different subclasses of NPs was determined by fluorescence microscopy after Hoechst 33342 staining combined with immunohistochemical staining for Sox2 and GFAP. As shown in Figure 6, the nuclear condensation in QNPs was slightly more in Model group than in Normal group although there was no statistical significance (*P* = 0.1501, Figure 6). The results suggested that CUS slightly enhanced the apoptosis of QNPs. On the contrary, EA treatment significantly inhibited the apoptosis of QNPs but not ANPs (*P* < 0.05, Figure 6). These results indicated that EA exerted some protective effects in QNPs to reverse the deleterious effects of CUS.

### 3.2. Discussion

The present study revealed that chronic EA treatment exerted significant antidepressant effects in a rat model of depression. Further, the mechanisms underlying antidepressant effects of EA were associated with preserving the QNPs from apoptosis and ameliorating the impaired ANPs proliferation in hippocampus.

Since the end of last century, Eastern and Western psychiatrists have implied the antidepressant-like and anxiolytic-like effects of EA in some independent clinical trials [38–43]. Besides, some animal studies further confirmed the antidepressant-like effects of EA [4, 44–46]. There are also some disagreements [47, 48]. The behavioural results of this experiment further confirmed that EA had some therapeutic efficacy in the rat model of depression, which is consistent with previous studies [45, 49]. FST is the most widely used tool for assessing depressive-like behaviours and antidepressant-like effects in rodents. Our results showed that EA significantly reduced the despair behaviour (immobility) and enhanced the active behaviour (such as, climbing) in rodent FST, which is indicative of antidepressant-like effects on behaviours. On the other hand, EPM tests anxiety-like behaviours in rodents, which are driven by the conflict between approaching and avoiding regions that are associated with aversive properties (i.e., the exposed spaces represented by the open arms). Similarly, EA showed anxiolytic-like effects in the EPM test. It significantly increased the percentage of entries and the time spent in the open arms. In addition, one-week EA treatment significantly improved the exploratory behaviour which was evaluated by measuring the number of rearings in OFT. However, EA and CUS did not show any effects on the horizontal distance which is an indicator of locomotor ability. Although it was consistent with several previous depression-model studies [50–52], EA exhibited positive effect on horizontal distance in other independent studies [53, 54]. This paradoxical result may be due to species heterogeneity, the use of different models of depression, or the different procedures of the OFT. Taken together, these results indicated that CUS-exposed rats develop significant anxiety-like and depressive-like behaviours but no changes of locomotor ability and EA exhibited antidepressant-like and anxiolytic-like effects in CUS rats.

In the current work, we also showed that EA was beneficial to the division of hippocampal NPs, which was consistent with the findings obtained by other studies demonstrating that EA promotes neurogenesis in different brain regions in rodent models of some brain disorders [4, 55–60]. Furthermore, our results indicated that EA majorly enhanced the proliferation of ANPs within DG of adult hippocampus. This suggested that EA was similar to the chemical antidepressant fluoxetine, which also increases the proliferation of ANPs but not QNPs [10]. On the other hand, many studies have confirmed that several kinds of stress can diminish the proliferation of NPs in adult hippocampus [61–66]. But it was uncovered that the defined subclass of NPs is sensitive to the stress. Our results simultaneously indicated that CUS has deleterious effects on the proliferation of both ANPs and QNPs. As shown in the published paper, the proliferation of NPs is the major contribution to the neurogenesis in adult hippocampus. Hence, the decreased proliferation of NPs induced by CUS will be manifested later as the declined neurogenesis, that is, a decrease in the birth of new neurons [67]. A recent published paper gives direct evidence that the decreased neurogenesis is implicated in the pathogenesis of anxiety and depression [68]. On the contrary, EA made the opposite effects; namely, it upregulated the proliferation of ANPs. That may be the underlying mechanism of antidepressant-like effects of EA. But, the molecular mechanisms of EA-induced signalling in the nervous system still warrant further investigation.

In addition, many intriguing reports have shown an association between major depression and selective and persistent loss of hippocampal volume; besides the decreased proliferation of NPs, overt death of hippocampal neural cells could cause this loss [69, 70]. Furthermore, a few number of papers have hinted that EA is protective and it promotes the survival of neural cells [71, 72]. Our recent published paper also presented the fact that EA protected astrocytes against atrophy; simultaneously, CUS significantly induced astrocyte atrophy [27]. Then, does the same effect also occur within the NPs? In this experiment, we did not observe significantly increased apoptosis of NPs in CUS rats, but apoptosis of QNPs was raised slightly in comparison to normal rats. The results hinted that CUS may be detrimental to the survival of NPs, especially QNPs. Moreover, we also observed the protective effect of EA on NPs. And, it majorly protected QNPs against apoptosis. The results further confirmed the beneficial effects of EA on hippocampal NPs.

Finally, the mechanisms underlying the contribution to proliferation and the effect against apoptosis toward NPs are far from being clarified. But based on a published study by us, one possible mechanism is that EA might interfere with the hippocampal microenvironment and enhance the activation of ERK signaling pathways, which are involved in both proliferation and antiapoptosis effects [73].

## 4. Conclusions

In summary, the present study demonstrated that CUS (4 weeks) induced significantly depressive-like and anxiety-like behaviours. CUS was deleterious to hippocampal NPs, in particular, inhibited the proliferation of NPs, and might enhance the apoptosis of QNPs. Furthermore, EA alleviated the depressive-like and anxiety-like behaviours induced by CUS and was directed against the deleterious effect of CUS on hippocampal NPs; that is to say, it promoted the proliferation of ANPs and protected QNPs against apoptosis.

## Abbreviations

EA: Electroacupuncture
NPs: Neural progenitors
DG: Dentate gyrus
QNPs: Quiescent neural progenitors
ANPs: Amplifying neural progenitors
CUS: Chronic unpredictable stress
FST: Forced swimming test
EPM: Elevated plus-maze
OFT: Open field test
BrdU: 5-Bromo-2-deoxyuridine.
